# Sepsis and multiple organ dysfunction in burn

**DOI:** 10.1186/cc12957

**Published:** 2013-11-05

**Authors:** Sabrina F Henrich, Tatiana H Rech, Iuri C Wawrzeniak, Rafael B Moraes, Karen F Prado, Marcos C Boniatti, Denise Matter, Lívia Biason, Geisiano Custódio, Roberto Ritter

**Affiliations:** 1 Unidade de Terapia Intensiva, Hospital de Clínicas de Porto Alegre, Brazil

## Background

Advances in the treatment of burns have reduced mortality rates and improved quality of life of victims. However, the most frequent complication is infection [1]. Thermal injury over 20% of the body surface area may lead to conditions similar to SIRS, as in septic shock. Beyond the extent of body surface area burned, which causes structural changes in skin coverage, other factors lead to infectious complications in burned patients: immunosuppression resulting from thermal injury, the possibility of gastrointestinal bacterial translocation, prolonged hospitalization, the use of devices and surgical procedures related to the burned areas [2,3]. C-reactive protein (CRP) is a known marker of infection and sepsis in patients admitted to the ICU.

## Materials and methods

CRP was measured in a cohort of 18 critically ill mechanically ventilated victims of a fire disaster in the city of Santa Maria, Brazil, on 27 January 2013, admitted to the ICU of the Hospital de Clínicas de Porto Alegre. The patients were divided into groups according to CPR levels, group 1 (CPR ≤190 mg/l) and group 2 (CRP >190 mg/l), and the Mann-Whitney test was performed to compare groups according to mortality, length of ICU and hospital stay, presence of sepsis and SOFA score on days 1, 3 and 7.

## Results

Six patients were male and the mean age was 23.1 ± 4.5 years. No differences were detected when patients were compared according to mortality (group 1: 0% vs. group 2: 11.1%; *P *= 0.48), length of ICU stay (group 1: 11 ± 8 days vs. group 2: 17.2 ± 9.7 days; *P *= 0.237) or length of hospital stay (group 1: 16.4 ± 8.5 days vs. group 2: 20.6 ± 10.2 days; *P *= 0.408). CPR levels were not associated with the development of sepsis (group 1: 50% vs. group 2: 80%; *P *= 0.321). The SOFA score was not significantly different between groups on day 1 and day 3 (day 1 - group 1: 4.6 ± 2 vs. group 2: 4.5 ± 2.7; *P *= 0.740; day 3 - group 1: 3.2 ± 2.7 vs. group 2: 5.8 ± 4.2; *P *= 0.203). However, the SOFA score was significantly higher on day 7 in group 2 (day 7 - group 1: 3 ± 0.7 vs. group 2: 5.8 ± 1.9; *P *= 0.017).

## Conclusions

CRP was not a good marker of sepsis and multiple organ dysfunction in this cohort of burned patients, possible due to the intense inflammatory response associated with burns.

